# Editorial

**Published:** 1865-09

**Authors:** 


### Editorial.

MEDICAL SCHOOLS IN CHICAGO.—Before the next issue of the *Examiner* will have reached its readers, the time for commencing the regular Annual Courses of Lectures in the medical colleges of this City, will have arrived. By the Annual Announcement of the Chicago Medical College, we learn that the next lecture term will commence on the *first Monday* in October, and continue, as usual, until the first Tuesday in March following. All the chairs in the Faculty are well filled; the means of illustration, both in the laboratory and museum, are ample; and the general course of instruction much more extensive and complete, than in most of the medical colleges in this country. It is also one of the first medical schools in this county, that has made daily *Hospital Clinical* instruction a necessary part of its course. Its intimate connection with the Mercy Hospital, gives it all the facilities in this respect that can be desired, or made available for the benefit of the student. Professor H. A. Johnson, President of the Faculty, who is spending the present season in Europe, will return before the opening of the lecture term, and bring with him some valuable additional means of illustration, particularly in the departments of anatomy, physiology, pathology, and microscopy. All further information needed, can be obtained by perusing the Announcement, or by addressing the Secretary of the Faculty, Professor E. Andrews, 101 Washington Street, Chicago, Ill.

Not having received a copy of the Annual Announcement of the Rush Medical College for 1865 and 1866, we can give no particular information concerning the coming term in that institution. We presume, however, it will be much the same as in former years.

---

### DEATHS.

Professor D. L. McGugin, of Keokuk, Iowa.—The death of this most faithful and enlightened member of our profession, was duly announced to us in the resolutions below, copied from the local papers of that city. Dr. McGugin had been, several years, one of the most influential members of the Faculty of the Medical Department of the Iowa University; and an active and highly esteemed member of the American Medical Association. He was universally respected as a physician, a citizen, and a Christian:—

Death of Dr. D. L. McGugin.—Pursuant to a call, the Keokuk Medical Society, together with the members of the medical fraternity of Keokuk, met at the office of the Secretary.

Present—Drs. Knowles, Seaton, Taylor, Cleaver, Harvey, McDonald, McIntosh, Sanford, Davis, Curtis, and Carpenter.

The meeting was called to order, and Dr. Knowles selected to preside, who briefly stated the object of the meeting to be the adoption of measures appropriate to the funeral services of D. L. McGugin, M.D.

On motion, it was

*Resolved*, That the medical fraternity of the city be requested to attend the funeral in a body, and that the members wear the usual badge of mourning.

On motion, a committee of three, viz.:—Drs. Taylor, Harvey, and Cleaver were appointed to draft resolutions expressive of the sense of this meeting.

It was further

*Resolved*, That the proceedings of this meeting be published in the daily papers, and that the family of the deceased be furnished with a copy.

Society adjourned to meet at Dr. Cleaver's office Sunday, at

2 o'clock P,M., and proceed in a body to the residence of the deceased. F. KNOWLES, M.D., *Chairman.*
A. M. CARPENTER, M.D., *Secretary.*

---

*Whereas*, In the wisdom of Divine Providence, our professional brother, Professor D. L. McGugin, has been taken from our midst to the "home of his fathers," therefore, as a last tribute to his departed worth, as a citizen and member of our profession and expressive of the sense of the members thereof in this city, it is hereby

*Resolved*, That, in his loss, which we deeply feel, we have been deprived of our associations with one who ever sought the highest standard of scientific attainment, and who has felt that the chief aim of his life, next to his religion, was to advance the interest of the profession he so signally adorned for a period of more than a third of a century.

*Resolved*, That as a patriot, he having twice entered the service of the Government, when struggling with foreign and domestic foes, he was true and loyal in the highest degree, and set a glorious example, by his personal sacrifices, his earnestness, and zeal, that the present and future members of our fraternity may well emulate.

*Resolved*, That, as a friend and member of this Society, we have ever found our deceased brother faithful, just, and kind-hearted; liberal in his treatment and opinions of others; and never actuated by narrow, jealous, or unkind sentiments.

*Resolved*, That we tender our heartfelt sympathies to the family and relatives of the deceased, and more particularly to her who has shared with him the struggles, anxieties, aspirations, and honors of an eventful life, do we offer our sincere condolence.

---

DR. JAMES COUPER, OF NEW CASTLE, DELAWARE.—"Died, suddenly, at New Castle, Delaware, on the 12th instant, James Couper, M.D." Such was the brief announcement in a Philadelphia paper, that gave us the first intelligence of the departure of one of the best men in the ranks of our profession. When the first circulars were issued, in 1845, inviting a convention of delegates from the medical societies and colleges in the United States, preparatory to the formation of the American Medical Association, Dr. Couper was one of the first to respond cordially to the proposition.

He attended the Convention in New York, in May, 1846, and was made chairman of the committee on the preliminary education of students of medicine. At the succeeding meeting in Philadelphia, he made an able and interesting report, which was fully approved by the meeting. Up to the time of his death, he remained an active and highly respected member of the National Association. He was an excellent example of the good physician, the upright citizen, and the true Christian. We deeply sympathize with his large circle of mourning relatives and friends.

---

Dr. Melvin N. Rust, Surgeon of U. S. Vols.—We copy the following notice of this most amiable and talented young physician, from the City papers:—

Intelligence has been received of the death of Major Melvin N. Rust, a graduate in the Chicago Medical College at its last term. He died at Nashville, Tenn., August 13th, of bilious dysentery, after an illness of some four or five days, and was only twenty-four years of age. Since his graduation, Dr. Rust has held the position of surgeon of the 154th Illinois Volunteer Infantry, and, at the time of his death, was chief surgeon of the brigade to which his regiment belonged, and surgeon in charge of the post hospital at Nashville. He had, for so young a man, a large and warm circle of friends; and possessed a kind heart, the most gentlemenly qualities, and far more than ordinary ability and industry, and gave every evidence that he would assume a high rank in his profession. A special meeting of the Faculty of the Chicago Medical College was held this afternoon, at which the death of Major Rust was announced, regarding which the following preamble and resolutions were unanimously adopted:—

*Whereas*, It has pleased Almighty God to remove from our midst Dr. Melvin N. Rust, our friend and former pupil, a member of the graduating class of 1864-5; therefore be it

*Resolved*, That in the death of Dr. Rust we feel the loss of a personal friend, and of one who gave unusual promise of future success in his profession, and of usefulness in the community, and who, by his gentlemanly qualities and marked ability as a student, secured alike our admiration and personal friendship.

*Resolved*, That with the numerous friends, and especially with his relatives and immediate family, we deeply sympathize in the loss they and we have sustained.

*Resolved*, That a copy of these resolutions be sent to the family of Dr. Rust, and that their publication be requested in the CHICAGO MEDICAL EXAMINER, and each of the daily papers of the City.

---

AMERICAN DENTAL ASSOCIATION.—The regular Annual Meeting of this organization was held in this City recently. The number in attendance was large; the proceedings harmonious, interesting, and profitable; and the social intercourse exceedingly pleasant. Below we give a part of the admirable welcoming address of Dr. Allport, chairman of the committee of arrangements. And as the subject of Specialties and Specialists is attracting some attention, at the present time, we have also given the substance of the remarks made in response to a sentiment offered at one of the evening entertainments:—

*Mr. President, and Gentlemen of the American Dental Association:*—In bidding you welcome to the City of Chicago, on an occasion so interesting and auspicious to our Profession as the present, it may not be deemed inappropriate to the time and place of our meeting, to indulge in a brief retrospect of the past.

In the summer of 1859, twenty members of the dental profession met in convention at Niagara Falls, to consult together as to the expediency of forming a National Association upon the representative basis. All, I believe, who were present felt that if such an association could be formed, and should receive the sanction and coöperation of any considerable portion of the better class of practitioners, the best interests of the profession would be promoted, and great good result to the public.

The number of state or local societies, then existing, to send delegates to the annual meetings of an association of this kind, was so very limited, that but few even of the small number present felt at all sanguine of the ultimate success of such an enterprise. But in view of the many great and good results anticipated from such association, in case it should be crowned with success, and in the hope that the formation of local societies would be stimulated thereby, it was determined to take the initiatory steps for the organization, deferring final action until the following year.

At the appointed time, July 31st, 1860, *twenty-three* delegates only, of the various dental societies and colleges then existing, met in the City of Washington, and the American Dental Association was organized, and entered upon its work. Five years only have passed, and from what was a small and doubtful beginning, by the steady and well-directed efforts of those who were instrumental in its formation, this association has become one of the most successful enterprises of the profession, and one of the most useful and influential dental societies in the world.

Of the professional standing of those who have attended, as delegates, the annual meetings of this Association, and have been accustomed to take part in its proceedings, it is not necessary for me to speak. They are universally acknowledged as standing among the most scientific and successful operators of our time and country, and as belonging to the class *progressive.* The essays and discussions of the members of this Society have passed into the literature of our profession, and become a part of its history. In point of ability they have been by far the ablest and most instructive that have ever emanated from any body of dentists in our country, and will suffer little, if any, in comparison with the essays and discussions of the American Medical Association. Should this Association adjourn *sine die* to-day, and never hold another meeting, yet sufficient good has already been accomplished by it to fully vindicate the wisdom and foresight of those who first projected it, and have done the most to sustain it.

As we cast our mind's eye over the history of dentistry, and see how, in the last forty years, it has risen from a tinkering, catch-penny calling to the dignity of a noble profession, in whose ranks may be found men of high moral and scientific culture, commanding alike the confidence and respect of the educated and refined, we can but attribute much of this progress and pleasing result to the influence of our various local and national associations. All of these associations have had their influence for good, and are important, but none is so well calculated, in every respect, to allay that spirit of jealousy and distrust in each other—that fatal millstone around the neck of all selfish communities—none so well calculated to strengthen the bond of a common interest and brotherhood, that should bind the profession of the East with the West, the North with that of the South, and make all to feel their mutual dependence upon each other as a representative National Association. In it are embodied the principles that underlie the structure of our National Government, which has demonstrated to the world that, for the protection of a community of interests, or for the development of resources, whether material or *mental*, no organization or government is so strong as that based upon the principles of a representative Republic.

You are all familiar with the advantages of association and combined labor in the various avocations of life, no matter whether it be in mental or physical labors. You assemble here to-day, as delegates and members from different parts of our extended land, and in the discussions here elicited you will find new illustrations of the old familiar truths that "knowledge is power," "union is strength," and "in a multitude of counsellors there is wisdom." By the contact of mind with mind, both will be strengthened, and embryo ideas and theories will be developed into full maturity: "There is a magnetism in such contact, full of creative energy. Flint and steel are passive in themselves, but clash them together and they give out fire, and brightness dazzles upon the sight. The positive and negative poles of a battery never come together without a *result.* By friction of different mental organizations together, an idea is evolved, a new law is discovered, a new creation is added to the wealth of knowledge, and the long-coming rays of a new truth, like those of a far-off star in the laboratory of heaven, reach and illumine the world."

Gentlemen, this Society was organized for a purpose. Its mission is not yet fulfilled. It has a great work to do—a destiny to accomplish. The men who were instrumental in its formation were not discouraged because it was so small and unpromising at first. They knew that in the profession it had a strong and vigorous mother to nurse it, and that its growth was certain, but they did not expect it to mature so rapidly. Nor will those who are now engaged in it be so elated by its unexpected success as to allow it to sink under that supine and careless indifference, which so often follows prosperity. Its course will be onward and upward—its motto, Union and Progress.

---

REMARKS OF DR. N. S. DAVIS, President of the American Medical Association, at an entertainment given by him, at his residence, to the Members of the Americal Dental Association, in response to a toast offered by Dr. C. W. SPAULDING, President of the Association, July 27, 1865:—

"To the President of the American Medical Association; Medicine, Surgery, and Dentistry—departments of a common science, their disciples should constitute a common brotherhood."

Dr. N. S. DAVIS, being generally called for, responded substantially as follows:—

*Gentlemen and Ladies:*—I am not only happy to be honored with your company this evening, but cheerfully respond to the sentiment just expressed by your honored President. That medicine, surgery, and dentistry are actually practical departments of a common science, very few will be disposed to deny. I say *common science*, in deference to popular custom. It would be more proper, however, to use the plural form of expression; for what is generally styled *medical science* is really an aggregation of many sciences, and their cultivation with direct reference to the prevention and alleviation of human suffering. The science of medicine, popularly so called, consists of facts and principles selected from every department of natural science, philosophy, psychology, political and social economy, and their application to the elucidation of the causes, nature, and treatment of such diseases, deformities, and injuries as are liable to afflict our race. Hence the student of medicine, in its general sense, is emphatically a student of nature. And not only so, but he studies the broad fields of nature for the highest and noblest of temporal objects, namely, to qualify himself for mitigating or relieving the imperfections, deformities, and diseases of his fellow men; whether they occur in the teeth, the organs of special sense, the extremities, or the more vital organs within the body.

Medicine, surgery, and dentistry are all based upon anatomy, physiology, pathology, and materia medica. Without anatomy none of you, as dentists, can know either the structure of a single tooth or its connections with the jaw, gums, bloodvessels, nerves, &c. Without physiology, none of you could know the natural uses and influences of the several parts just named, or the relations of the teeth to the whole processes of digestion, assimilation, and nutrition. As pathology bears the same relation to organized structures in an imperfect or diseased condition, as physiology does to them in the natural, so without a knowledge of it, neither the physician, surgeon, or dentist could know anything of the origin, nature, and tendencies of the diseases and defects he professes to treat. The materia medica, in its full scope, includes everything that can be made useful in the mitigation or removal of any of the ills to which man is liable. The gold that fills a cavity in a tooth, the wash that soothes an irritated gum, and the instruments used for adjusting both, are as much a part of the armamenta or materia medica, as are the pills and powders administered by the physician. These four branches of medical study are fundamental, and no man can do full justice, practically, to the most limited specialty without a thorough knowledge of them all.

Every member of your Association will acknowledge that a dentist should certainly understand the composition, structure, and mode of development of the teeth, together with the causes that render their development defective, or induce in them disease and decay. But in a single tooth you have three out of the five primary forms of living, structural organization, namely, the fibrous, vascular, and nervous, with the peculiar arrangement of inorganic matter to give it solidity.

A knowledge of these structures, whether chemically, anatomically, or microscopically, involves a knowledge of the same structures in all other parts of the body. To understand the development of a tooth and its appendages, from materials selected from the blood, involves a knowledge of the blood itself, and all the laws that govern the intricate processes of assimilation, nutrition, and disintegration in living structures generally. The same remark applies with equal emphasis to the causes of imperfections and diseases of the dental organs, and the means for remedying them. Indeed, there is not a living atom of our physical organization so isolated that a knowledge of its structure, nutrition, disintegration, and various morbid conditions, can be obtained, without developing all the essential facts and principles of anatomy, physiology, and pathology.

So far, therefore, as dentistry is a science, as distinguished from a mere mechanical art, it rests on the same foundation, and necessarily involves the same series of studies as all other departments of medicine and surgery.

Of late, much has been said about specialties and specialists; and there is, at this day, especially in our country, a manifest tendency to favor the multiplication of both. We are told that instead of a few leading divisions of medicine and surgery, we should have almost as many specialties as there are important organs in the human body; and that every individual member of the profession should devote himself to the study and treatment of some one class of diseases, or the diseases of some one organ or apparatus of organs. By thus concentrating attention upon a limited number of diseases or injuries, it is claimed that greater skill will be acquired in their treatment; and more advancement in our knowledge of their nature and tendencies. Division of labor and concentration of attention, is said to have been the parent of most of the discoveries and improvements of modern times. It is further claimed that the whole field of medical and surgical sciences, with their practical application, is so extensive that it is impossible for one man to so master the whole, as to properly qualify himself for the practice of all its dedartments.

This process of reasoning is plausible, and to a limited extent true. It is true that in all the mere mechanical arts, the greater the division of labor, and the more perfectly each man is restricted to a certain series of movements, the greater will be the skill and accuracy acquired in their performance.

The Dentist who restricts his work entirely to the processes of filling teeth, may possibly acquire greater skill in that particular work, (provided he has enough of it to do,) than he would if he filled teeth, extracted teeth, fitted artificial teeth, &c.

The Surgeon who restricts himself entirely to the more important and delicate operations on the eye, or the ear, or the bloodvessels, may acquire greater dexterity in those particular operations, than if he attended to the whole field of operative surgery. But the rule applies properly, only to such operative procedures as are essentially mechanical; and cannot be extended to the treatment of diseases of particular organs, without causing more mischief than good. The reasons are obvious to every enlightened and reflecting mind.

*First*, the various organs of the human body are not so many isolated parts, the functions and diseases of which have no influence upon each other, but they are so intimately connected and mutually dependent, that not a single morbid impression can be made on one organ that will not exert a modifying influence on all the rest.

The same heart sends the blood to every organ and structure in the body. The same nervous centres radiate the delicate threads that are to impart sensibility or command motion to the remotest parts of our organization. And the same vital properties pervade every living atom. Every link in the chain of actions constituting digestion, assimilation, nutrition, disintegration, and excretion is so connected that not one can be broken or marred without embarrassing the action of the whole. Hence, it is literally impossible to comprehend the nature, tendencies, and results of the diseases of one organ without studying their influence on all the others and *vice versa*. Hence there can be no such thing as specialism proper, in the study of pathology or the nature of diseases. The whole field must be studied before any one of its parts can be fully understood.

*Second*, the circumstances of civilized communities ever have, and probably ever will, prevent any extensive division of practical medicine and surgery into specialties, except in a few large cities and densely populated towns. Much the larger part of the inhabitants of every country occupy the rural districts, where they are obliged to send from one to ten or fifteen miles for a practitioner of any kind. Now, suppose each member of the profession was devoting himself to such special diseases as he imagined to be most in consonance with his taste, and a farmer living five miles from his market place or business centre, should be attacked with acute pain in his side, and send for Dr. A. on the supposition that his attack was inflammation of the lungs. After ten or twelve hours delay, Dr. A. arrives, but finds on examination that the pain is certainly located below the diaphragm, and of course no disease of the lungs. Inasmuch as the diseases of the lungs and throat constitute his SPECIALTY, the case does not belong to him and he retires. From six to ten hours more are spent in getting Dr. B., whose specialty embraces the diseases of the abdominal viscera or digestive organs. But, on examination of the case, Dr. B. finds the acute pain in the side of his patient to be neuralgic and dependent on the disease of the spinal cord or its membranes, at the origin of the nerves affected. This places the case out of the range of Dr. B.'s practice, and makes it necessary to send ten miles to a neighboring town for Dr. C., whose specialty embraces diseases of the cerebro-spinal nervous system.

Again, suppose the farmer, instead of having been attacked with a pain in his side, had fallen from a load of hay and broken his leg, and wounded an artery in his hand.

A messenger is sent in great haste to the nearest town for Surgeon A., who comes, perhaps, with a new and improved fracture box, and dresses the broken leg with much skill and dexterity. This done, he asks for his fee, and takes his hat to depart. Hold, Doctor, exclaims the patient; you have dressed my leg, but are you going to leave me to bleed to death from this wound in my hand? Oh, my dear sir; blandly replies the Surgeon, that is not in my department. I attend only to fractures or injuries of the bones. For injuries and diseases of the bloodvessels, you must send for Surgeon B., in the adjoining town. Without multiplying illustrations, is it not obvious that specialties can be made practically beneficial only in populous towns; and even in them only to a limited degree? It may be said that the cases cited as illustrations are of the nature of *emergencies*, which every professional man must be prepared to treat, at least temporarily. But how is he to be prepared to treat such cases, even for the briefest practicable period, without having so studied the whole field of professional science and practice, as to be familiar with all its parts? If he has so studied it, what becomes of the argument or pretense that the field is so extensive that one man cannot keep himself master of the whole? The truth is, that the greater part of medical and surgical practice consists of emergencies. It is not one time in ten, that the physician or surgeon, when he starts to visit a patient for the first time, knows what kind of disease or injury he will find on his arrival. And he can neither satisfy an enlightened conscience nor do justice to a confiding community, unless he is always prepared to meet such emergencies, and treat whatever ills may be presented to him in a skillful and proper manner.

If a man, living in the country, is attacked with iritis or acute conjunctivo-cornitis, and suffers a permanent disorganization of the textures of the eye and loss of vision before he can possibly reach a reliable occulist, it is a very unsatisfactory apology for his family physician to allege that diseases of the eye constitute a *specialty* to which he has given little or no attention. Yet many such cases are constantly occurring throughout the country.

But, gentlemen, there is still another aspect of this subject worthy of a moment's thought. From the very nature of the laws that govern mental operations, exclusive practical attention to any one department of a general subject, tends to contract and bias the mind, by giving undue relative importance to one series of facts, while neglecting another series of equal real value. An evil of much greater magnitude, however, consists in the strong tendency of specialism to encourage incompleteness of professional education. During a connection with medical teaching for sixteen years, I have rarely found a student who on his final examination, proved himself ignorant of some important department, without alleging that he did not intend to practice that particular department, and consequently had paid less attention to it. Indeed, incompleteness of education leading to the adoption of partial and restricted views, and the universal tendency to neglect whatever is not intended to be turned directly to practical pecuniary advantage, constitutes the foundation of much of the evils that exist in the profession of our country.

You are, doubtless, ready to ask by this time, if I then oppose all specialties in medicine. By no means. There is a certain natural basis on which a limited number of specialties can be founded with great advantage; and which, indeed, develope themselves by the natural and inevitable course of circumstances. For instance, the diseases, deformities, and defects of the dental organs, involving no immediate danger to life, and requiring for the treatment of many of them, special mechanical manipulations, naturally, and almost necessarily, constitute a special department of surgery. A department, indeed, that should be regarded as equal in importance and dignity, and consequently requiring equal education with every other branch of the profession. Those conditions of the eye requiring delicate and dexterous operations are also most of them chronic, and allow the patient time to seek and obtain the services of men who have acquired more than ordinary skill in the performance of such operations. The same is true of those conditions requiring some of the most dangerous and difficult surgical operations upon other parts of the body; such as lithotomy, ovariotomy, tying large and deep-seated arteries for aneurism, &c. Hence it is eminently proper that in all large cities where the required opportunities are afforded, men should devote special attention to such departments. But this never can justify any class of medical men for contenting themselves with only a partial professional education.

Mr. President, with the last clause of the sentiment you have offered, I most cordially agree. That all educated and honorable physicians, surgeons, and dentists should constitute a "*common brotherhood*," is dictated alike by a community of interest, a similarity of education, and a common object, namely, the alleviation of human suffering and the prolongation of human life.

I am glad to see the prosperity of your Association and its truly national character. Like the American Medical Association, it constitutes a strong social, as well as professional link to bind all parts of our country indissolubly together. And permit me to add, that if the members of the legal and ecclesiastical professions, from the East, West, North, and South, would annually commingle with the same cordiality and liberal feeling, it would so perfectly bind the hearts of this great nation that *secession* would never again drench our loved land in human blood.

### ON THE DIAGNOSIS AND TREATMENT OF SCABIES.

Perhaps there is no disease, which has passed through so many phases, and caused so much dispute amongst savans, as the complaint now so well understood as scabies or the itch. It seems singular that a disease, apparently well understood by the Greeks and Romans, should, so late as the beginning of the present century, have been misunderstood, both as to its cause and progress. Yet the acarus, so well known now, for the first twenty years of the present century, was denied an existence by most practitioners.

But, on the other hand, a false impression is given the student by WILSON, when he leads us to suppose that, after a day's work, one may easily take from a patient 18 or 20 acari, and examine them at his leisure under the microscope. My experience has been, that in very few cases, not more than one in twenty, can the acarus be found, and then only by the greatest care and perseverance.

It must be remembered that JOHN HUNTER, an acute observer, never found an acarus, and many good dermatologists have had the same experience. During my studies, connected with the Boston Dispensary, under Dr. WHITE and Dr. DAMON, in more than 100 cases, I have only seen the acarus four times. For a general description of the acarus and his habitat, I refer to MCCALL ANDERSON, and will here only speak of the positions in which the acarus is most likely to be found.

The burrow of the acarus, first red, then dark and tortuous, and from four to five lines in length, is, of course, the diagnostic mark of scabies. As to the eruption, I do not think it can often be distinguished from others of a vesicular character. The first place which has been recommended as the site of burrows, is between the fingers. But a much more probable one, is the point where the ends of the fingers touch the palm when the hand is closed, as the acarus is carried to this point under the nail, after scratching. In males, the penis seems to be a favored locality, for reasons which it is not necessary to specify. In the hospitals of Vienna, this site is never overlooked. In infants and young children, the hips offer most promise, as it is here the hands of the nurse oftenest fall. In fact, the burrow is more likely to be found anywhere, in a perfect condition, than between the fingers, except in the earliest stages. The burrow having been found, if one wishes to explore for the "little stranger," let him open one of the most promising, at the end where a vesicle is just making its appearance, and if he finds *her*, he is extremely fortunate.

I have no doubt there are some cases of scabies, where the burrow cannot be found. But it must be remembered that the hands of artisans, who work in acrid substances, are liable to ecthymatous eruptions, so children who are badly or insufficiently fed. But it is better that the burrow should be found, before making a diagnosis of scabies, for a preparation which would be suitable for scabies (such as strong black soap, recommended abroad by physicians, and in this country by Dr. HARDY, of St. Louis,) would be inapplicable to cases of ecthyma or impetigo.

Having discovered the burrow of the acarus, the treatment is very simple. It is understood by all, that sulphur ointment is almost a specific. I generally unite

℞. Potassæ Carbonat., ---------------------- ʒj.

Sulphur, ------------------------------------ ʒij.

Adipis, ------------------------------------- ℥j.

As to the use of the black soap, the *savon vert* of Paris, the oil soap of this country, I am not in favor of it, except in cases of extreme filth, where, in process of time, we hope to see the cuticle. If the eruption of scabies is complicated with any other, showing a bad state of the blood, I think a mild soap, slightly caustic, is preferable and quite sufficient. The patient should remain in a warm bath an hour before applying the unguent. In all cases, I believe tr. ferri chlorid. should be prescribed as a tonic. In private practice, where a neater prescription is sometimes desirable, I would recommend as an unguent,

℞. Potass. Iodid, ------------------------- gr. x.
Adipis, -------------------------------- ℥j.

to be repeated two or three times. I believe it would be equally effectual with sulphur ointment. I am, Sir,

Very truly, yours,
E. A. P. BREWSTER, A.M., M.D.,
*Janesville, Wis.*

---

TRANSACTIONS OF ILLINOIS STATE MEDICAL SOCIETY.—The Annual Volumes of Transactions of our State Medical Society have been published, and copies mailed to all the members who have paid their annual assessment for the present year.

It is proper to mention, that only a small part of the members have paid; and consequently the treasury is, not only empty, but in debt to the extent of nearly two hundred dollars. If each member of the Society, who is in arrears for the assessment of the present year ($3), would promptly remit the amount to Dr. J. H. Hollister, of Chicago, Treasurer, it would at once settle the printer's bill, and secure to himself a copy of the Transactions, which is well worth the money. It contains 152

pages, and the report of Dr. Prince on Orthopedic Surgery, is alone of sufficient interest and value to make it desirable for every member of the profession.

---

American Medical Association.—Members desiring copies of the *Transactions* for 1865, must forward their subscriptions ($3) immediately, as the edition will be limited to the number of paid subscriptions.

WM. B. ATKINSON, *Permanent Sec'y.*

---

CITY MORTALITY FOR JUNE AND JULY, 1865.

DISEASES.

| | | | |
|---|---|---|---|
| Apoplexy, | 1 | Fever, Scarlet, | 3 |
| Accidents, | 4 | " Typhoid, | 4 |
| Bronchitis, | 1 | " Typhus, | 2 |
| Burned, | 1 | " Brain, | 2 |
| Congestion of Brain, | 6 | " Puerperal, | 1 |
| " " Lungs, | 2 | Heart Diseases, | 3 |
| Cholera Morbus, | 1 | Inflammation of Brain, | 7 |
| Cholera Infantum, | 1 | " " Bowels, | 2 |
| Consumption, | 18 | " " Lungs, | 3 |
| Croup, | 15 | Killed, | 2 |
| Cramps, | 12 | Liver Complaint, | 2 |
| Convulsions, | 10 | Measles, | 1 |
| Childbirth, | 5 | Old Age, | 4 |
| Cerebro-Spinal Meningitis, | 3 | Paralysis, | 1 |
| Drowned, | 11 | Rheumatism, | 1 |
| Diphtheria, | 4 | Suicide, | 2 |
| Dropsy, | 9 | Small-Pox, | 3 |
| Diarrhœa, | 2 | Stillborn, | 9 |
| Dysentery, | 1 | Summer Complaint, | 9 |
| Erysipelas, | 1 | Teething, | 6 |
| Epilepsy, | 1 | Unknown, | 4 |
| Epileptic Convulsions, | 1 | Water on Brain, | 7 |
| Found Dead, | 2 | | |
| Fever, Lung, | 2 | Total, | 195 |
| " Nervous, | 1 | | |

Ages of the Deceased.—Under 5 years, 77; over 5 and under 10 years, 23; over 10 and under 20, 10; over 20, and under 30, 16; over 30 and under 40, 24; over 40 and under 50, 12; over 50 and under 60, 6; over 60 and under 70, 5; over 70 and under 80, 3; over 80 and under 90, 3; stillborn, 9; unknown, 7. Total, 195.

NATIVITIES.

| | | | | | |
|---|---|---|---|---|---|
| Chicago, | 106 | Scotland, | 3 | Other States, | 19 |
| Canada, | 5 | Sweden, | 1 | Unknown, | 2 |
| Colored, | 2 | Germany, | 17 | | |
| England, | 10 | Norway, | 5 | Total, | 195 |
| Ireland, | 24 | Illinois, | 1 | | |

DIVISIONS OF THE CITY.

North, ......... 41 | South, ........ 70 | West, ......... 84 | Total, ......... 195

Total number of deaths in June, 1864, ........................................ 262

DISEASES.

| | | | |
|---|---|---|---|
| Asthma, | 3 | Intemperance, | 5 |
| Apoplexy, | 5 | Intermittent Fever, | 1 |
| Accident, | 6 | Inflammation of Brain, | 2 |
| Burned, | 3 | Inflammation of Bowels, | 10 |
| Bright's disease, | 2 | Inflammation of Lungs, | 4 |
| Brain fever, | 4 | Liver Complaint, | 1 |
| Bilious Fever, | 2 | Measles, | 5 |
| Congestion of Brain, | 9 | Marasmus, | 2 |
| Congestion of Lungs, | 4 | Nervous Fever, | 1 |
| Cholera morbus, | 10 | Old Age, | 8 |
| Cholera infantum, | 7 | Paralysis, | 1 |
| Cold, | 1 | Puerpural Fever, | 1 |
| Calculus, | 1 | Suicide, | 1 |
| Cancer of Stomach, | 2 | Stillborn, | 6 |
| Cancer of Head,, | 1 | Summer Complaint, | 94 |
| Consumption, | 24 | Sun Stroke, | 2 |
| Cramps, | 35 | Spasms, | 1 |
| Croup, | 3 | Sore Throat, | 1 |
| Convulsions, | 16 | Scarlet Fever, | 25 |
| Childbirth, | 6 | Scalded, | 1 |
| Cerebro-Spinal Meningitis, | 1 | Typhoid Fever, | 13 |
| Drowned, | 1 | Typhus Fever, | 1 |
| Diphtheria, | 7 | Teething, | 22 |
| Dropsy, | 8 | Tumor, | 1 |
| Dysentery, | 20 | Whooping Cough, | 2 |
| Diarrhœa, | 9 | Unknown, | 18 |
| Erysipelas, | 1 | | |
| Found dead, | 3 | Total, | 426 |
| Flux, | 1 | | |
| Heart Disease, | 4 | Same month last year, | 426 |

Ages of the Deceased. — Under 5 years, 271; over 5 and under 10 years, 14; over 10 and under 20, 15; over 20 and under 30, 26; over 30 and under 40, 32; over 40 and under 50, 16; over 50 and under 60, 10; over 60 and under 70, 17; over 70 and under 80, 7; over 80 and under 90, 3; stillborn, 8; unknown, 15. Total, 426.

NATIVITIES.

| | | | | | |
|---|---|---|---|---|---|
| Chicago, | 273 | Holland, | 1 | Sweden, | 3 |
| Other States, | 55 | Ireland, | 28 | Wales, | 2 |
| Bohemia, | 1 | Norway, | 10 | Unknown, | 3 |
| Canada, | 1 | Poland, | 1 | | |
| England, | 7 | Prussia, | 1 | Total, | 426 |
| Germany, | 24 | Scotland, | 4 | | |

DIVISIONS OF THE CITY.

North, .........126 | South, .........130 | West, .........170 | Total, ......... 426

---

We call attention to the following notice, and invite an early answer.—[Ed. Examiner.]

MEDICAL PRACTICE WANTED.

An ex-surgeon of the United States Volunteers, who was in the practice of medicine in the South-west before the commencement of the Rebellion, desires to purchase the whole or onehalf of a good medical practice, or to hear of a good location. Please address *Surgeon*, in care of Doctor N. S. Davis, No. 23 Washington Street, Chicago, Ill.

---

TRANSACTIONS OF THE AMERICAN MEDICAL ASSOCIATION.

PHILADELPHIA, Feb. 6, 1865.

*Dear Sir*,—As there are various methods by which the volume may be sent, inform me which you prefer. If by mail, please forward *thirty-two cents* in post-office stamps, that your postage may be prepaid.

Very respectfully,
CASPAR WISTER,
*Treasurer American Med. Association.*
No. 1303 Arch Street.

The following volumes are for sale:—
Proceedings of the Meeting of Organization, 50 cents.
(Vols. I., II., III., IV., and VI., are out of print.)
Vols. V., VII., VIII., and IX., *if taken collectively*, $5 for the set. If singly, $2 apiece.
Vols. X., XI., XII., XIII., and XIV. at $2 each.
Vol. XV. at $3.
Vol. XVI. in press.

---

ANTISEPTIC INJECTION FOR THE CADAVER.—Dr. S. W. Wetmore, in the *Buffalo Medical and Surgical Journal*, recommends as the best agent for this purpose, the arsenite of soda. Boil 12 ounces crystallized carb. of soda and 8 ounces arsenious acid in 32 fluid ounces of water for one hour. Then add water to make three quarts of the solution, which will be ample for two subjects. He injects into the femoral artery. Bodies thus injected remain free from vermin. The preparation "not only acts as a disinfectant, but an antiseptic, destroying all virus, rendering the tissues firm, the muscles retaining their color and solidity, prohibiting decomposition and offensive odors." Dr. W. is so well satisfied with the efficacy of the solution as to affirm that "it renders all former preparations obsolete." There is no risk in pricking the hand either from animal virus or from the arsenic. Arsenic alone will not answer the purpose.—After the injection the body should be left undisturbed for 12 hours or longer. The bloodvessels will then be found empty, and should be injected with melted tallow colored with Venetian red, or some other substance, for the purpose of distending them and facilitating dissection. Death from hemorrhage is most favorable for the preservation of the body. Sudden death generally favors decomposition. In sunstroke, the body often has a putrid odor before life is extinct. These facts have a practical bearing in connection with the embalming of soldiers dying in the service.

---

ANÆSTHESIA BY ELECTRICITY.—"The sedative effects of the constant current," says M. Remak, (who is now experimenting at La Charité,) "when the current is very feeble, are exceedingly interesting. To produce such effects, in fact, the current must never excite painful sensations. The sedative action produced by this current differs from that of other sedatives; and it may be employed when, for various reasons, the use of opium, belladonna, etc., is objectionable. One of the most striking instances in which the current is of service is in removing the increased sensibility of an inflamed part. If, in such a case, a positive electrode, of sufficiently extended surface, be applied over the seat of inflammation, and the negative electrode at a distant part of the body, we shall find, in the course of five or ten minutes, that the sensibility of the part has greatly diminished. Thus, for example, in a case of very painful inflammation of the elbow or wrist, we place the positive pole over the brachial plexus, and the other over the scapula; and we find the pain is soon lessened. Lately, in the presence of MM. Claude Bernard, Velpeau, and Beau, I applied the current in the case of a man who ten days before had struck his knee, and suffered great pain at the inner border of the patella. The pain was so great that the patient could not walk except with his knee bent. I placed the positive electrode over the crural nerve below Poupart's ligament and the other pole over the extensors of the leg. In a few minutes we observed that the joint became less painful, and the extension of the limb more easily performed. The patient was completely cured by three applications of the remedy. Let me remark to all those who would repeat my experiment, that the curative effect depends upon the surface of the elements of the pile; that is to say, that piles composed of small elements must be absolutely rejected."—*British Medical Journal* and *Dublin Medical Press.*

---

PREMIUM OFFERED.—Dr. T. C. Brinsmade, of Troy, N. Y., offers a premium of $100 for the best essay on medical and vital statistics, with a plan for hospital reports and records of private practice, and a draft of a registration law. The essay is to be handed to the Committee of the New York State Medical Society on Prize Essays by the 15th of December next.

